# UniProt: the universal protein knowledgebase in 2021

**DOI:** 10.1093/nar/gkaa1100

**Published:** 2020-11-25

**Authors:** The UniProt Consortium, Alex Bateman, Maria-Jesus Martin, Sandra Orchard, Michele Magrane, Rahat Agivetova, Shadab Ahmad, Emanuele Alpi, Emily H Bowler-Barnett, Ramona Britto, Borisas Bursteinas, Hema Bye-A-Jee, Ray Coetzee, Austra Cukura, Alan Da Silva, Paul Denny, Tunca Dogan, ThankGod Ebenezer, Jun Fan, Leyla Garcia Castro, Penelope Garmiri, George Georghiou, Leonardo Gonzales, Emma Hatton-Ellis, Abdulrahman Hussein, Alexandr Ignatchenko, Giuseppe Insana, Rizwan Ishtiaq, Petteri Jokinen, Vishal Joshi, Dushyanth Jyothi, Antonia Lock, Rodrigo Lopez, Aurelien Luciani, Jie Luo, Yvonne Lussi, Alistair MacDougall, Fabio Madeira, Mahdi Mahmoudy, Manuela Menchi, Alok Mishra, Katie Moulang, Andrew Nightingale, Carla Susana Oliveira, Sangya Pundir, Guoying Qi, Shriya Raj, Daniel Rice, Milagros Rodriguez Lopez, Rabie Saidi, Joseph Sampson, Tony Sawford, Elena Speretta, Edward Turner, Nidhi Tyagi, Preethi Vasudev, Vladimir Volynkin, Kate Warner, Xavier Watkins, Rossana Zaru, Hermann Zellner, Alan Bridge, Sylvain Poux, Nicole Redaschi, Lucila Aimo, Ghislaine Argoud-Puy, Andrea Auchincloss, Kristian Axelsen, Parit Bansal, Delphine Baratin, Marie-Claude Blatter, Jerven Bolleman, Emmanuel Boutet, Lionel Breuza, Cristina Casals-Casas, Edouard de Castro, Kamal Chikh Echioukh, Elisabeth Coudert, Beatrice Cuche, Mikael Doche, Dolnide Dornevil, Anne Estreicher, Maria Livia Famiglietti, Marc Feuermann, Elisabeth Gasteiger, Sebastien Gehant, Vivienne Gerritsen, Arnaud Gos, Nadine Gruaz-Gumowski, Ursula Hinz, Chantal Hulo, Nevila Hyka-Nouspikel, Florence Jungo, Guillaume Keller, Arnaud Kerhornou, Vicente Lara, Philippe Le Mercier, Damien Lieberherr, Thierry Lombardot, Xavier Martin, Patrick Masson, Anne Morgat, Teresa Batista Neto, Salvo Paesano, Ivo Pedruzzi, Sandrine Pilbout, Lucille Pourcel, Monica Pozzato, Manuela Pruess, Catherine Rivoire, Christian Sigrist, Karin Sonesson, Andre Stutz, Shyamala Sundaram, Michael Tognolli, Laure Verbregue, Cathy H Wu, Cecilia N Arighi, Leslie Arminski, Chuming Chen, Yongxing Chen, John S Garavelli, Hongzhan Huang, Kati Laiho, Peter McGarvey, Darren A Natale, Karen Ross, C R Vinayaka, Qinghua Wang, Yuqi Wang, Lai-Su Yeh, Jian Zhang, Patrick Ruch, Douglas Teodoro

**Affiliations:** European Molecular Biology Laboratory, European Bioinformatics Institute (EMBL-EBI), Wellcome Genome Campus, Hinxton CB10 1SD, UK; Protein Information Resource, Georgetown University Medical Center, 3300 Whitehaven Street NW, Suite 1200, Washington, DC 20007, USA; Protein Information Resource, University of Delaware, Ammon-Pinizzotto Biopharmaceutical Innovation Building, Suite 147, 590 Avenue 1743, Newark, DE 19713, USA; SIB Swiss Institute of Bioinformatics, Centre Medical Universitaire, 1 rue Michel Servet, CH-1211 Geneva 4, Switzerland

## Abstract

The aim of the UniProt Knowledgebase is to provide users with a comprehensive, high-quality and freely accessible set of protein sequences annotated with functional information. In this article, we describe significant updates that we have made over the last two years to the resource. The number of sequences in UniProtKB has risen to approximately 190 million, despite continued work to reduce sequence redundancy at the proteome level. We have adopted new methods of assessing proteome completeness and quality. We continue to extract detailed annotations from the literature to add to reviewed entries and supplement these in unreviewed entries with annotations provided by automated systems such as the newly implemented Association-Rule-Based Annotator (ARBA). We have developed a credit-based publication submission interface to allow the community to contribute publications and annotations to UniProt entries. We describe how UniProtKB responded to the COVID-19 pandemic through expert curation of relevant entries that were rapidly made available to the research community through a dedicated portal. UniProt resources are available under a CC-BY (4.0) license via the web at https://www.uniprot.org/.

## INTRODUCTION

The UniProt databases exist to support biological and biomedical research by providing a complete compendium of all known protein sequence data linked to a summary of the experimentally verified, or computationally predicted, functional information about that protein. The UniProt Knowledgebase (UniProtKB) combines reviewed UniProtKB/Swiss-Prot entries, to which data have been added by our expert biocuration team, with the unreviewed UniProtKB/TrEMBL entries that are annotated by automated systems. The UniRef databases cluster sequence sets at various levels of sequence identity and the UniProt Archive (UniParc) delivers a complete set of known sequences, including historical obsolete sequences. UniProt additionally integrates, interprets, and standardizes data from multiple selected resources to add biological knowledge and associated metadata to protein records and acts as a central hub from which users can link out to 180 other resources. In recognition of the quality of our data, and the service we provide, UniProt was recognised as an ELIXIR Core Data Resource in 2017 (1) and received the CoreTrustSeal certification in 2020. The data resource fully supports the Findable, Accessible, Interoperable and Reusable (FAIR) data principles (2), for example by making data available in a number of community recognised formats, such as text, XML and RDF and via Application Programming Interfaces (API)s and File Transfer Protocol (FTP) downloads, providing stable and traceable identifiers for protein sequence and protein sequence features and by fully evidencing our data sources throughout. We have also reviewed and updated our data licencing policies.

UniProt is continually evolving to meet new challenges while still working to capture all available protein sequence data and to curate the ever-increasing amount of functional data described in the scientific literature. In our last update published in this journal in 2019 (3), we described how we are responding to the growth in microbial protein sequence records, largely derived from high-quality metagenomic assembled genomes. These will increasingly be added to by large-scale eukaryotic sequencing programs, such as the Darwin Tree of Life (www.darwintreeoflife.org) and Earth Biogenome (www.earthbiogenome.org) projects. Collectively, these have already resulted in the number of entries contained in UniProtKB growing by >65 million records, an increase of >50% in just 2 years. As the volume of sequence data continues to grow, we will continue to explore different ways to ensure database sustainability and scalability whilst still providing the best possible service to our user community.

## PROGRESS AND NEW DEVELOPMENTS

### Growth of sequence records in UniProt

**Figure 1.**
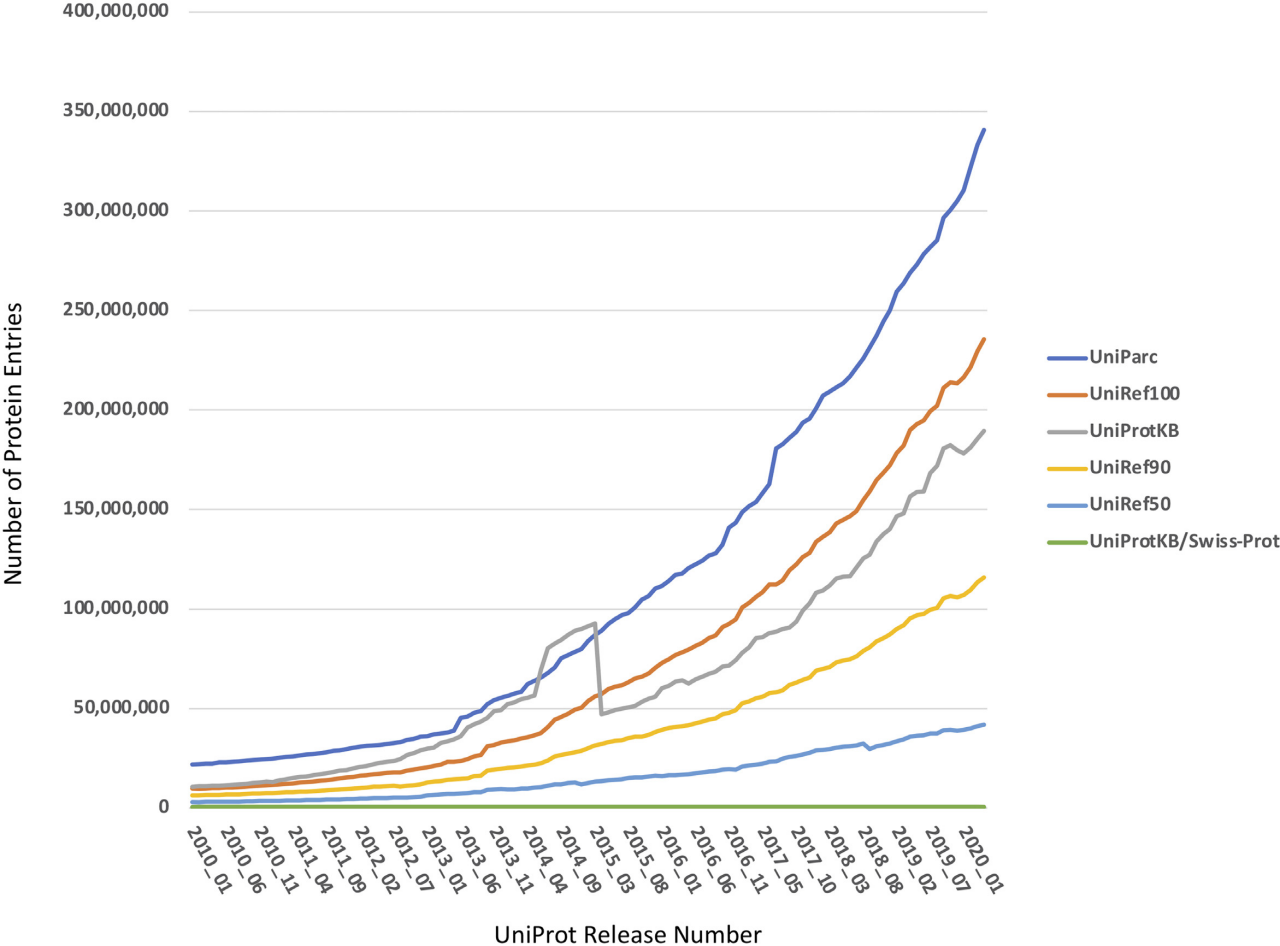
Growth in the number of entries in the UniProt databases over the last decade.

UniProt release 2020_04 contains over 189 million sequence records (Figure 1), with >292 000 proteomes, the complete set of proteins believed to be expressed by an organism, originating from completely sequenced viral, bacterial, archaeal and eukaryotic genomes available through the UniProtKB Proteomes portal (https://www.uniprot.org/proteomes/). The majority of these proteomes continue to be based on the translation of genome sequence submissions to the INSDC source databases—ENA, GenBank and the DDBJ (4)—supplemented by genomes sequenced and/or annotated by groups such as Ensembl (5), NCBI RefSeq (6), Vectorbase (7) and WormBase ParaSite (8). Viral proteomes are manually checked and verified and periodically added to the database.

UniProt proteomes provide the set of proteins currently believed to be expressed by an organism. Previously, this dataset only consisted of complete proteomes derived from fully sequenced genomes. This has now been updated to enable the user to access a wider set of proteomes with different quality and completeness metrics. To enable researchers to evaluate proteome completeness and expected gene content, we have adopted the BUSCO (Benchmarking Universal Single-Copy Orthologs) scoring method for vertebrate, arthropod, fungal, and prokaryotic organisms on the Proteomes portal, in addition to providing details of species and the protein count. BUSCO v3 (9) identifies complete, duplicated, fragmented, and potentially missing genes by comparison to a defined set of near-universal single copy orthologs. As a result of our adoption of the BUSCO algorithm, a number of incomplete and poor-quality proteomes were identified and removed from UniProtKB.

**Figure 2.**
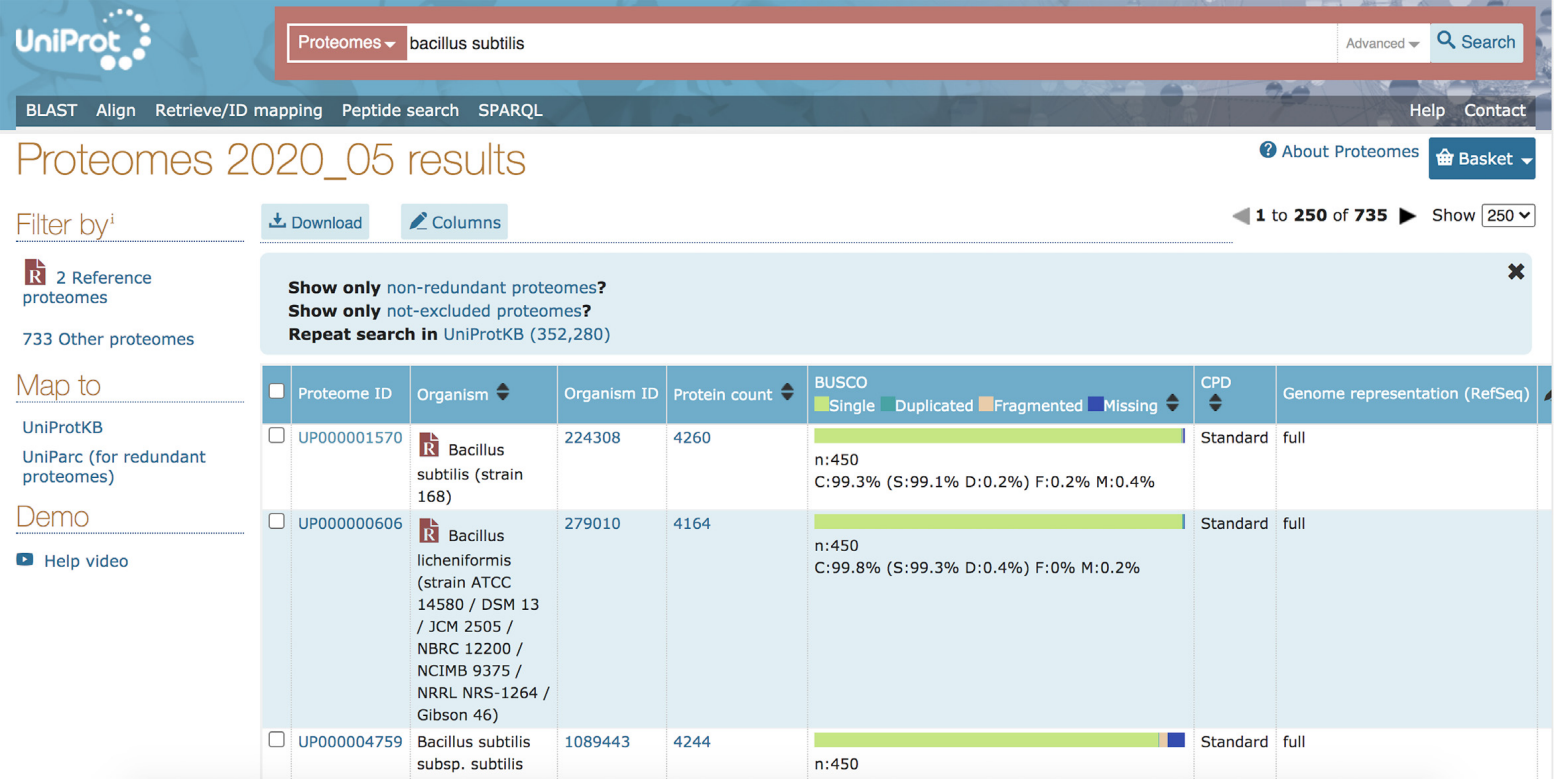
*Bacillus subtilis* proteomes viewed on the Proteomes webpage with BUSCO and CPD scores. The left-hand panel suggests further option by which the user could filter the data, for example by only selecting reference proteomes.

The Proteomes webpage has been redesigned to enable users to view full details of their proteome(s) of interest in a single table view (Figure 2). We also display the results of the ‘Complete Proteome Detector’ (CPD), an in-house algorithm, which statistically evaluates the completeness and quality of each proteome by directly comparing it to those of a group of at least three closely taxonomically related species. The CPD classifies each proteome as either ‘standard’, ‘close to standard’ or an ‘outlier’, according to protein count vs. the standard distribution of protein count expected for completeness in comparison to a cluster of closely related organisms (https://www.uniprot.org/help/assessing_proteomes). We additionally provide the assessment of the genome assembly status imported from the source of the genome assembly and annotation (e.g. Ensembl or RefSeq). Users may sort by BUSCO or CPD score and/or filter by membership of the Reference Proteome set (∼7% of total proteomes) A Reference Proteome is one that has been selected either by the research community or by computational clustering (10) as providing the best annotated proteome in their cluster and is stably maintained as the chosen representative unless a higher quality proteome is identified in that cluster. UniProt Proteome pages now also provide a link to download a one-to-one protein set for the corresponding number of unique genes found in the genome. For each gene, a single protein sequence is algorithmically chosen from the proteome. This allows users to get a gene-centric subset of representative proteins for a given genome, as opposed to the full proteome which includes all proteins (e.g. including isoforms) that map to the genome.

As previously described (3), we continue to remove almost identical proteomes of the same species from UniProtKB (https://www.uniprot.org/help/proteome_redundancy), leaving only a relevant representative proteome in that section of the database. The redundant proteome sequences are available through UniParc to researchers and stable proteome identifiers (of the form UPXXXXXXXXX, where Xs are integers) are maintained for each redundant proteome to ensure findability.

UniRef protein sequence clusters facilitate sequence similarity searches, functional annotation, gene prediction, and genome and proteome comparisons. We have adopted the MMseqs2 algorithm to improve the speed of UniRef production (11), decreasing the time taken to perform UniRef50 clustering from four weeks to 60 hours, and improved procedures to compute proteome clusters and identify representative proteomes, pan proteomes, and core and accessory proteomes.

### Expert curation

**Figure 3.**
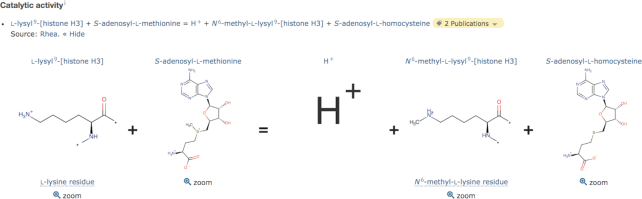
Catalytic activity comment and Rhea reaction visualized by the reaction graphic for the histone-lysine *N*-methyltransferase EHMT2 (UniProtKB:Q96KQ7).

The evaluation of experimental data published in the scientific literature, and summarizing key points of biological relevance in the appropriate reviewed UniProtKB/Swiss-Prot record, is fundamental to the operation of the UniProt database. The functional information extracted from the literature is added both in the form of human readable summaries and via structured vocabularies, such as the Gene Ontology (GO) (12). The curators also work to improve the computational accessibility of UniProt records, for example updating and replacing the existing textual descriptions of biochemical reactions in UniProtKB using the Rhea knowledgebase of biochemical reactions (13). Rhea (14) uses the chemical ontology ChEBI (Chemical Entities of Biological Interest) (15) to describe reaction participants, their chemical structures and chemical transformations. The complete ChEBI ontology is indexed to support hierarchical searches on the UniProt website so that a user searching on a top-level term such as ‘phosphatidylinositol’ (CHEBI:28874) will find reactions involving derivatives such as 1-phosphatidyl-1D-myo-inositol 3,4,5-trisphosphate (CHEBI:16618). Users can also search using Rhea identifiers as well as identifiers, names, synonyms and chemical structures (encoded as InChIKeys) from ChEBI. A user-friendly visualization for chemical reactions has also been developed to enable Rhea reactions to be viewed on the website within the appropriate UniProt entry (Figure 3). UniProtKB/Swiss-Prot currently includes annotations for 8,058 unique Rhea reactions, which feature in 220 003 distinct UniProtKB/Swiss-Prot protein records (39.1% of all UniProtKB/Swiss-Prot records are annotated with Rhea) (release 2020_04 of 12 August 2020). The annotation of pseudoenzymes has also been reviewed and updated, in collaboration with experts in this field (16).

To further ensure our data is both human-readable and also computationally-tractable and continues to adhere to the FAIR principles (2), we are working to standardize the representation of all existing UniProt data on the functional impact of human variation. UniProtKB/Swiss-Prot has curated over 81 000 variants in 13,000 human protein sequence records from peer-reviewed literature, annotated with information on the functional impact and clinical significance, when known. Over 30 000 of these variants have been associated with Mendelian diseases. Clinical significance is evaluated using the guidelines of the American College of Medical Genetics and Genomics and the Association for Molecular Pathology (ACMG-AMP) (17) and ClinGen tools such as the pathogenicity calculator (18), with all clinical interpretations routinely submitted to ClinVar to promote reuse (19).

**Figure 4.**
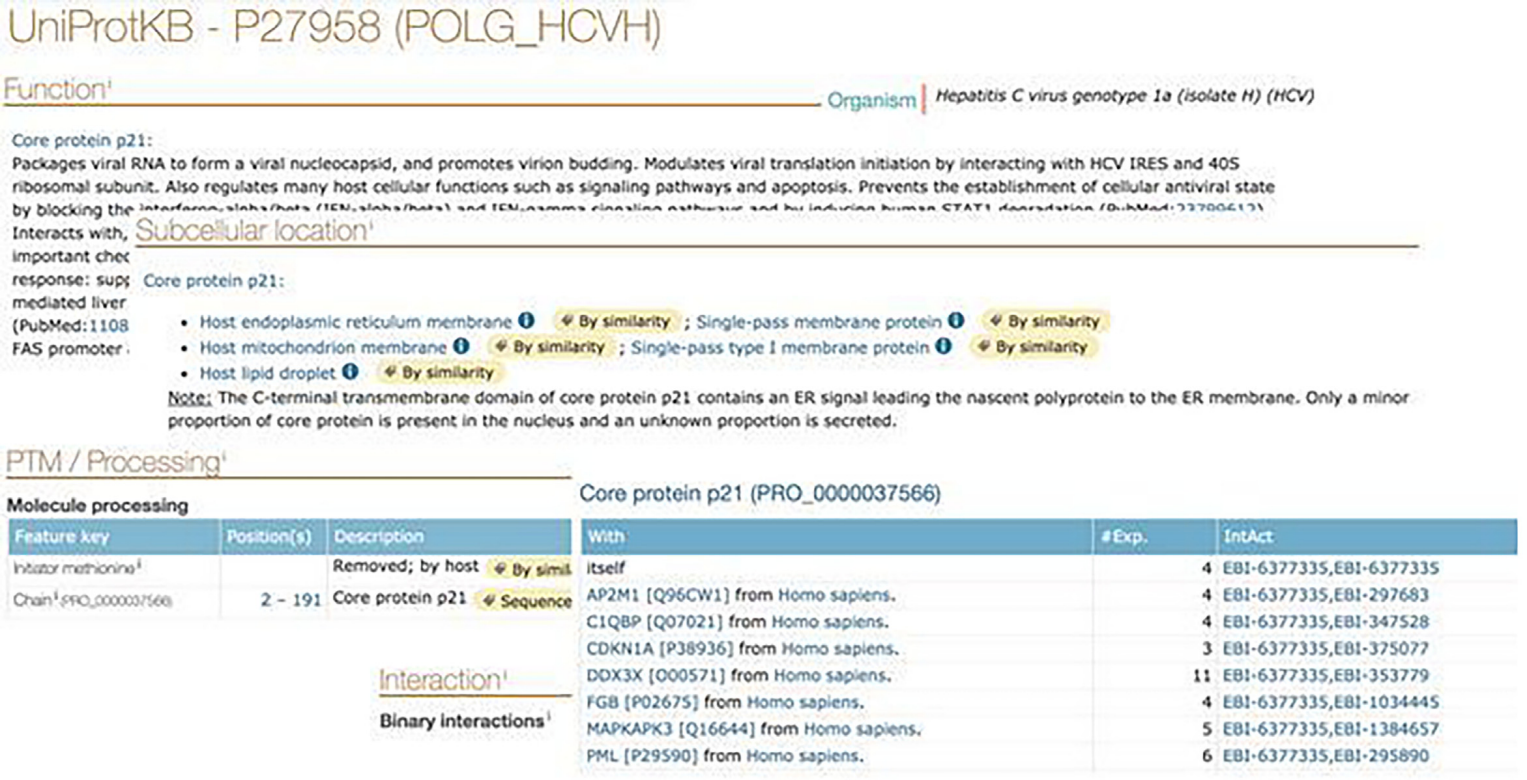
Information extracted from an entry describing Hepatitis C viral protein (UniProtKB:P27958) highlighting annotation added at the processed mature chain level, describing the p21 core protein. (PRO_0000037566).

In addition to the increased use of structured vocabularies to enhance accessibility to UniProtKB records, we have also improved the presentation of the information within each entry. The vast majority of multi-exon genes undergo alternative splicing to produce a variety of splice isoform proteins, which can potentially increase the functional diversity of proteins. Currently at least 95% of human genes are believed to be alternatively spliced (20,21) resulting in an estimated 75 000 distinct protein coding sequences. Post-translational proteolytic cleavage, where proteins are cleaved to remove some additional amino acid(s) or portion of protein, creates yet more mature amino-acid chains as a single polyprotein may generate multiple bioactive proteins or peptides. We have adapted our data model to capture machine-readable functional annotations for specific isoforms and polyprotein cleavage products, and now provide such knowledge for >5000 protein sequence entries. This enables the users to mine the data to identify cases where alternative protein sequences generated from the same gene have different functions. UniProtKB provides stable identifiers to both isoforms and to post-translationally cleaved chains which are used by many of our collaborators to identify these specific protein sequences in their own resources. This, in turn, has allowed us to further improve our mapping of imported data, for example binary protein interactions imported from the IMEx Consortium of molecular interaction databases are now displayed at the specific isoform/post-processed chain level. The representation of isoform-specific annotations and sequence features has been enhanced in the website to facilitate the exploration of this information in UniProt (Figure 4).

### Responding to the COVID-19 pandemic

The broad expert knowledge of our curation team enables us to quickly respond to areas of new and rapidly expanding biology. Severe Acute Respiratory Syndrome Coronavirus 2 (SARS-CoV-2) was identified as the cause of the 2019–2020 COVID-19 viral outbreak and ensuing pandemic. Its genome sequence was first made publicly available by the International Nucleotide Sequence Database Collaboration (INSDC) on 10 January 2020 (accession number MN908947). UniProt curators specializing in viral proteomes rapidly annotated the proteins encoded by this viral genome, first by similarity to other closely related coronaviruses, and subsequently by updating relevant entries with experimental data as soon as this was published. A pre-release dataset was made publicly available, first as text files on the UniProt FTP site, followed by the launch of a dedicated COVID-19 disease portal in March 2020 (https://covid-19.uniprot.org), providing the latest available pre-release UniProtKB data for the SARS-CoV-2 coronavirus and other viral and human entries relating to the COVID-19 outbreak. As the pandemic has progressed, these entries have been prioritized for further update to support global research efforts, with information relating to a more granular understanding of protein functionality, 3D-structure, binary interactions, and complex formation being added when either published or deposited in collaborating resources. The COVID portal is currently being updated more frequently than the standard 8-weekly UniProt release cycle to ensure the research community accesses these data in a timely manner.

The COVID-19 disease portal is a prototype of our intention to provide disease-centric access points to the wealth of data contained in UniProtKB records. Another recent example of a disease-focused curation effort has been the update of records relating to Alzheimer's disease, including proteins containing a disease-related amino-acid variant, their interacting partners and model organism proteins important for our understanding of disease initiation and progression. This work has been supported by clinical researchers active in the field who contributed to a number of workshops held in both the USA and UK and have suggested key protein targets for focused curation (22,23) and provided valuable user input into how this data should be accessed.

### Automatic annotation

The ever-increasing amount of genomic data arising from current sequencing projects means that the proportion of unreviewed records in UniProtKB/TrEMBL describing largely predicted proteins represents by far the largest, and most rapidly growing, section of UniProtKB. These unreviewed records are enriched with functional annotation by systems using the protein classification tool InterPro (24), which classifies sequences at superfamily, family and subfamily levels, and predicts the occurrence of functional domains and important sites. In UniProtKB/TrEMBL entries, domains predicted by the InterPro member databases PROSITE, SMART or Pfam are used to automatically provide domain annotations. The semi-automated rule-based computational annotation UniRule system (25) annotates experimentally uncharacterized proteins based on similarity to known experimentally characterized proteins, adding properties, such as protein name, functional annotation, catalytic activity, pathway, GO terms and subcellular location. We continue to increase the number of UniRules used for annotation and this set has now grown to 6768 (release 2020_04) rules in total.

To complement the expert guided process of creating UniRules, we have recently (release 2020_04) introduced the Association-Rule-Based Annotator (ARBA), a multiclass, self-training annotation system for automatic classification and annotation of UniProtKB proteins (3). This replaces the previous rule-based SAAS system. ARBA is trained on UniProtKB/Swiss-Prot, then uses rule mining techniques to generate concise annotation models with the highest representativeness and coverage based on the properties of InterPro group membership and taxonomy. ARBA employs a data exclusion set that censors data not suitable for computational annotation (such as specific biophysical or chemical properties) and generates human-readable rules for each release which are made available at https://www.uniprot.org/arba/. 22 894 ARBA rules were used to annotate 87 325 890 proteins in release 2020_04, increasing the combined coverage of the rule-based annotation systems from 35% to 49% in UniProtKB/TrEMBL. Sequence feature predictions are currently excluded from annotation by ARBA. Additionally, in release 2020_04, more than 15 million uncharacterized protein names have been improved using InterPro member database signatures, updating their name to ‘domain X containing protein’ following the International Protein Nomenclature Guidelines (https://www.uniprot.org/docs/International_Protein_Nomenclature_Guidelines.pdf). For example, the uncharacterized Western lowland gorilla protein UniProtKB:G3RLC3 has now been renamed ‘SH2 domain-containing protein’ giving biological information to the user. This system includes adding names based on domains of unknown function (e.g. UniProtKB:A0A009EMH9 DUF4372 domain-containing protein) as, although not immediately informative, it enables protein grouping thus improving the chances of eventually assigning a function to that domain. All automatic annotations are labelled with their evidence/source.

As previously described (3), UniFIRE is an open-source Java-based framework and tool developed to apply the UniProt annotation rules on given protein sequences and provided by UniProt to share our knowledge in computational annotation and our rule-based systems (https://gitlab.ebi.ac.uk/uniprot-public/unifire). This system is freely available for groups to use for in-house protein annotation projects (26) or to contribute their own rules in the URML (UniProt Rule Markup Language) format which may be reused for the annotation of UniProtKB entries.

The automatic annotation systems described above require the presence of an ordered region of protein that can be recognized as a domain or provide a signature of family membership which has been identified by an InterPro member database. However, over 20% of unreviewed proteins in UniProt do not contain any InterPro signature regions, and many InterPro signatures are not associated with transferable annotation. The largest part of missing annotation seems to derive from intrinsically disordered (ID) protein regions, therefore we have collaborated with the MobiDB-lite resource to provide a consensus-based prediction of long disorder (27). The method uses eight different predictors to derive a consensus which is then filtered for spurious short predictions. 109 144 661 predictions of regions of disorder, plus those described as ‘Basic’, ‘Polar’, ‘Acidic’, ‘Polyampholyte’ and ‘Pro-’ or ’Cys-rich’ have been added to 37 286 893 unreviewed entries and it is planned to also import these annotations into the appropriate UniProtKB/Swiss-Prot entries.

### Data Integration

UniProtKB integrates large-scale datasets, mapping these data onto the appropriate protein sequence records and displaying the mappings via the ProtVista visualisation tool (28) and downloadable via FTP and APIs (29). Clinically relevant sources of variation (e.g. 100K genomes, gnomAD and ClinVar SNPs) are mapped to protein features and variants using a pre-calculated mapping of the genomic coordinates for the amino acids at the beginning and end of each exon and the conversion of UniProt position annotations to their genomic coordinates (30). Functional positional annotations from the UniProt human reference proteome are now being mapped to the corresponding genomic coordinates on the GRCh38 version of the human genome for each release of UniProt. These mappings are also available as BED files or as part of a UniProt genomic track hub and can be downloaded from the UniProt FTP site (www.uniprot.org/downloads). Aligning variants to protein features, such as functional domains and active sites, ligand binding sites and PTMs in the UniProt record, can provide mechanistic insights into how specific variants can lead to disease or resistance to a drug or to a pathogen.

UniProt additionally integrates and visualizes unique and non-unique peptides identified by mass spectrometry proteomic data deposited through the ProteomeXchange Consortium (31) (e.g. PeptideAtlas (32), MassIVE (33) and jPOST (34)) and other large-scale initiatives (CPTAC (35), ProteomicsDB (36), MaxQB (37), ETD and CTDP (38)). Peptide identifications can be taken as evidence that a protein has been validated (PE = 1) using a variation of the HPP guidelines (in brief, at least two unique peptides of seven amino acids or more or, for proteins where this cannot be achieved due to sequence constraints, one unique peptide of ten amino acids or more has been mapped to a protein). UniProt also provides the new format PEFF (PSI Extended FASTA Format) proposed by the HUPO-PSI (Human Proteome Organization-Proteomics Standard Initiative) for sequence databases (39) to be used by sequence search engines and other associated tools (e.g. spectral libraries search tools). The UniProt PEFF format currently represents variation and sequence data and is available through the Proteins API service (https://www.ebi.ac.uk/proteins/api/doc/) for 31 species.

### Community curation in UniProt

**Figure 5.**
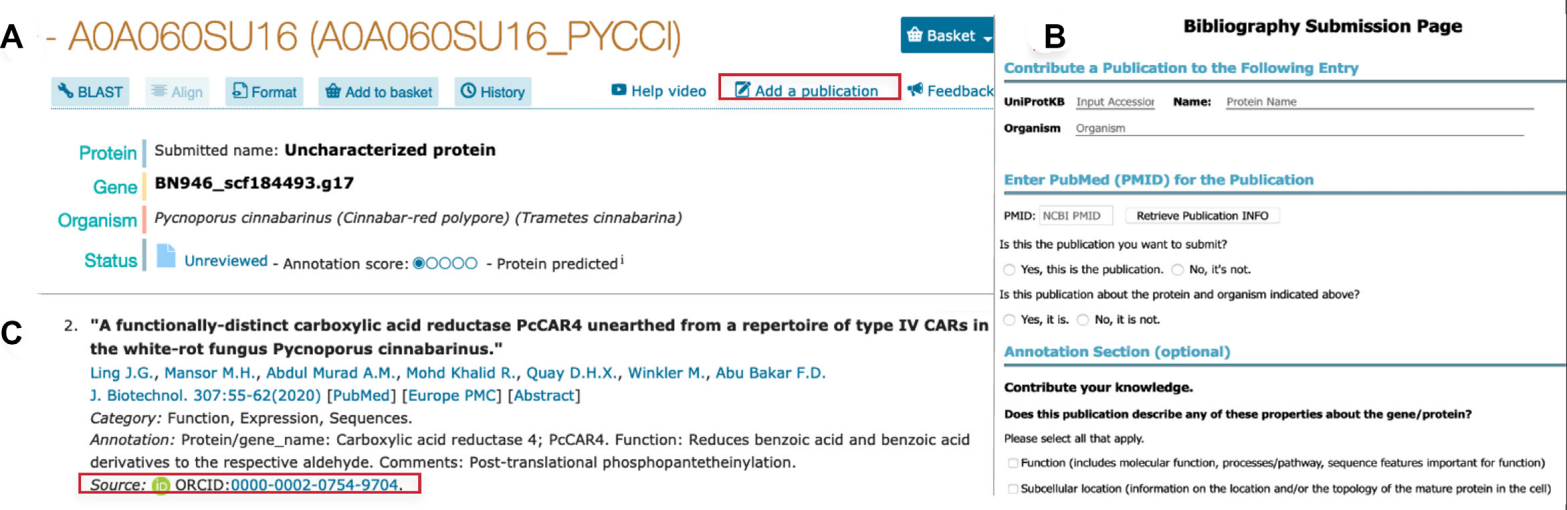
Community contribution. (i) Use ‘Add a publication’ functionality (red box) in the UniProtKB entry. (ii) Partial snapshot of the submission form, a sample available here: https://community.uniprot.org/bbsub/sampleform.html. (iii) After submission and review the publication and information are displayed in the relevant UniProtKB entry with attribution to submitter (red box) in a future public release.

UniProt users have always actively engaged with us and provide important feedback to the resource. The significant number of requests we receive through the Help Desk for articles and annotations to be added to protein entries prompted the development of the ‘Community submission’ page, where researchers are able to add articles that they deem relevant to an entry and provide optional basic annotation by selecting the topics relevant to each paper from a controlled list and/or adding short statements about protein name, function, and disease, as described in the publication. Contributors are asked to supply their ORCID (https://orcid.org/), a researcher personal ID, which is used to both validate that the submission is genuine and to give credit to the submitter for their work (Figure 5). This enables us to leverage the scientific community as a resource for enhancing our curated content, emulating a model already adopted by a number of model organism databases, such as WormBase (40), PomBase (41) and FlyBase (42).

Submissions are minimally checked by an experienced curator before being added to the Publications section of the record. A tracker tool has also been developed (https://community.uniprot.org/bbsub/bbsubinfo.html) to enable users to access and search this wealth of additional bibliography. Researchers are encouraged to add relevant publications to entries of interest to them. Since we started the pilot in release 2019_08 we have seen a continuing increase in user submissions. As of release 2020_04 there have been 674 submissions relating to 424 publications and 557 entries, from 149 unique users (https://community.uniprot.org/bbsub/STATS.html).

### Website updates

Users are at the center of the UniProt website design and development process. We follow a user-centered design process, conducting regular workshops, user testing, surveys and user research activities involving many users worldwide with varied research backgrounds and use cases. We are currently redesigning the website to take advantage of new technologies and paradigms in web development and welcome the UniProt user community to participate in user feedback and testing activities by contacting us at help@uniprot.org and contributing your user stories and workflows.

**Figure 6.**
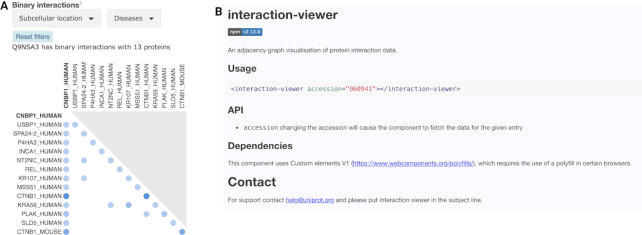
(**A**) The UniProtKB interaction viewer as seen in entry UniProtKB:Q9NSA3, the beta-catenin-interacting protein 1. (**B**) The interaction viewer reusable web component in the Nightingale library.

As part of the redesign process, we have redeveloped both the UniProt interaction viewer (Figure 6A) and ProtVista viewer, which graphically represents protein sequence features, such as domains, sites, post-translational modifications and variants, and structures. These are now part of the ‘Nightingale’ visualization web component library (https://ebi-webcomponents.github.io/nightingale/#/) and are publicly available as lightweight, flexible, and modular components that can be more easily extended with new features, modified and implemented by users in their own resources (Figure 6B). The ProtVista viewer has already been implemented by the Open Targets (43) and the Pharos (44) databases of unstudied and understudied drug targets amongst others.

## DATA AVAILABILITY

Due to the ever-increasing number of sequence records UniProt is processing with every release cycle, as of release 2020_01 (26 February 2020), UniProt releases are now published every eight weeks. This gives our production team the time required to complete data import, proteome redundancy removal, data checking, integration of external data and automatic annotation of unreviewed records prior to starting the release process.

In addition to providing customizable views and downloads in a range of formats via the website, and file sets at the FTP site (www.uniprot.org/downloads), UniProt supplies users with a number of different options for computational access to the data (www.uniprot.org/help/programmatic_access). These include the website RESTful Application Programming Interface (API), stable URLs that can be bookmarked, linked, and reused, the Proteins extended REST API providing genomic coordinates of UniProtKB sequences and annotations imported and mapped from large-scale data imports (29), and the SPARQL API that allows users to perform complex queries across all UniProt data and also other resources that provide a SPARQL endpoint, such as DisGeNET (45), Bgee (46), or Wikidata (47). The Proteins API has recently been extended to serve the HUPO Proteomics Standards Initiative Extended FASTA Format (PEFF) for the proteomics community which enables more metadata, such as detail of amino-acid variants in the FASTA file header section (39).

### Outreach and training

The training of our users to make the best use of our data continues to be of critical importance to the UniProt Consortium. In order to access our global user base, we are increasingly moving away from classroom style training towards use of distance learning techniques. We regularly run webinars, which range from basic introductions to the use of the web pages aimed at new users and students, to more specialist sessions on, for example, use of the API or 3D structural representation in UniProtKB. The webinars are recorded and are subsequently made available online (https://www.ebi.ac.uk/training/online/) where they are supported by related online training materials and YouTube videos (https://www.youtube.com/user/uniprotvideos/). In order to reach new communities, the webinars are widely advertised through social media forums such as Twitter (@uniprot) and Facebook (https://www.facebook.com/uniprot.org/) as well as established mailing lists.

## CONCLUSIONS

UniProt continues to play its pivotal role in the fields of biology and biomedicine, collecting, standardizing and organizing knowledge of proteins and their functions to create a reference framework for multiscale biomedical data integration and analysis. Organisms are being routinely sequenced at the whole genome level, and eukaryotic, prokaryotic, and metagenomic sequencing projects are all contributing to the increased diversity of sequence data in the UniProt databases. It is of increasing importance that our automatic annotation pipelines continue to develop in parallel to ensure that these unreviewed genomes, the vast majority of which are not being experimentally studied at the protein level, are richly and comprehensively annotated with functional information. Expert curation of those proteins biochemically characterized remains a key focus of our activities, to both inform on these well-studied entities and also to act as template entries for information transfer to proteins in related species. As the complexity and depth of our value-added data increases, we are exploring new ways to present the data to users and will continue to serve the community with new and improved website access designed to improve and enhance the user experience and upgraded programmatic access, with ease of use always a priority.

We greatly value the feedback and annotation updates from our user community. Please send your feedback and suggestions to the e-mail address help@uniprot.org or via the contact link on the UniProt website.
